# ‘A lot of small things make a difference’. Mental health and strategies of coping during the COVID‐19 pandemic

**DOI:** 10.1111/hex.13416

**Published:** 2021-12-22

**Authors:** Emma C. Halliday, Vivien Holt, Koser Khan, Fiona Ward, Paula Wheeler, Gill Sadler

**Affiliations:** ^1^ Division of Health Research, Faculty of Health & Medicine Lancaster University Lancaster UK

**Keywords:** coping strategies, diary method, mental health, pandemic

## Abstract

**Introduction:**

The social and economic consequences of COVID‐19 have the potential to affect individuals and populations through different pathways (e.g., bereavement, loss of social interaction).

**Objective:**

This study adopted a solicited diary method to understand how mental health was affected during England's first lockdown. We also considered the experiences of diary keeping during a pandemic from the perspective of public participants.

**Methods:**

Fifteen adults older than 18 years of age were recruited from northwest England. Diarists completed semistructured online diaries for 8 weeks, which was combined with weekly calls. A focus group captured participants' experiences of diary keeping.

**Findings:**

Four key factors influenced mental health, which fluctuated over time and in relation to diarists' situations. These concerned navigating virus risk, loss of social connections and control and constrictions of the domestic space. Diarists also enacted a range of strategies to cope with the pandemic. This included support from social networks, engagement with natural environments, establishing normality, finding meaning and taking affirmative action.

**Conclusion:**

Use of diary methods provided insights into the lived experiences of the early months of a global pandemic. As well as contributing evidence on its mental health effects, diarists' accounts illuminated considerable resourcefulness and strategies of coping with positive effects for well‐being. While diary keeping can also have therapeutic benefits during adversity, ethical and practical issues need to be considered, which include the emotional nature of diary keeping.

**Public Contribution:**

Members of the public were involved in interpretation of data as well as critiquing the overall diary method used in the study.

## 1 INTRODUCTION

The impact of the COVID‐19 pandemic on mental health is recognized to be considerable, with potential to affect individuals and populations through a variety of pathways.^1^, ^2^ Direct effects on mental health may result from grief and bereavement,^3^ as well as from the loss of social interactions and relationships due to social distancing and lockdown policies.^4^ Indirectly, it is also likely that there will be longer‐term consequences associated with financial difficulties and job losses.^5^

As a result of social distancing measures, the qualitative research community has adapted to online methods to capture lived experiences of the pandemic.^6^, ^7^ Diary studies have been proposed as a method to elicit day‐to‐day insights about the pandemic's social effects, where nonparticipant observation is not feasible.^8^ In the context of previous crises, they have been used to reveal experiences of recovery after flooding^9^ and an agricultural crisis,^10^ informing policy responses about the longer‐term support required by communities.

The solicited diary method, designed for research purposes (as opposed to private diary keeping), is recognized to have advantages.^11^ Temporally, they allow for the recording of changing experiences (e.g., over days, weeks or months).^12^ For some, diary writing may lead participants to reveal more private insights into situations that they are less willing to discuss in an interview context.^13^ Conversely, however, this aspect of diary keeping also raises ethical considerations.^14^, ^15^ Bartlett and Milligan point out that while diary keeping can have therapeutic and positive benefits, the recollection of negative situations also risks psychological distress.^16^

A growing body of COVID‐19 studies has adopted diary methods to explore the pandemic's effects on mental health^17^, ^18^ and social relationships^19^, ^20^, ^21^ among general adult populations^17^, ^19^, ^21^ and young people.^22^, ^23^ Collectively, these studies collecting data at multiple time points show how experiences fluctuate, with participants reporting, for instance, periods of positive well‐being as well as anxiety^18^ or greater loneliness during the week than at weekends.^21^ However, while these studies adopt the temporal aspects of diary keeping, they are primarily quantitative in design, providing less opportunity for participants to record their personal experiences. Two exceptions are diary studies of children and young people in Italy^22^ and the United Kingdom.^23^ While both studies highlight negative well‐being effects arising from isolation and anxiety, the qualitative accounts in these diaries also illuminate the ways in which individuals demonstrate resourcefulness and resilience in coping with the pandemic's effects.^22^

In this paper, we report on a diary study conducted with members of the public during the first wave of the COVID‐19 pandemic in northwest England. We focus specifically on its mental health effects, drawing attention to flux and diversity in these experiences, as well as the strategies used to cope with the pandemic. Finally, as few studies have adopted diary methods during crises, we reflect on the method from the perspective of public participants, with particular consideration given to its emotional and ethical aspects.

## 2 STUDY DESIGN AND METHODS

The study used a solicited diary design, adopting diary tools developed for use in previous crisis situations in England.^9^, ^10^ Additionally, we adopted a mixed‐methods approach combining diary keeping with weekly calls, previously found to aid diary completion as well as enhance the quality of data collected.^15^ As the study was exploratory, we did not formulate precise research questions in advance, but sought to broadly understand public experiences of the pandemic including its effects on diarists' everyday lives.

The following theories and concepts informed the findings presented. First, we drew on the World Health Organization's definition of mental health. This recognizes mental health as a ‘state of well‐being’ (which may be positive or negative), rather than only characterized by diagnosed conditions (e.g., anxiety, depression) and influenced by multiple factors.^24^ Second, the disaster management literature was used to identify coping strategies adopted by communities during previous crises (e.g., establishing normality, taking affirmative action, role of faith).^25^, ^26^ This helped to structure our analysis and interpretation of data but also enabled some comparison as to whether strategies adopted during the pandemic were similar or different to other crises.

Participants (referred to henceforth as ‘diarists’) were public advisers to the ‘Applied Research Collaboration North West Coast' (ARC NWC). The ARC NWC is a collaboration between five universities and a range of public/third sector organizations, and has a strong ethos of public involvement in research. Diarists played a dual role, principally as study participants, but were also involved in critiquing the diary method. All participants were over 18 years old and resident in northwest England. An invitation email was sent to the ARC NWC email list to all registered public advisers. Fifteen participants self‐selected to participate, and all completed the study. One further adviser initially agreed to participate, but withdrew before data collection commenced. Demographic and contextual information was collected using an anonymous monitoring form and at the first weekly call (Table 1).

**Table 1:** Overview of diarists participating (*N* = number of participants)

Age	*N*	Employment status	*N*	Gender	*N*
30–39	1	Self‐employed	2	Female	10
40–49	2	Retired	4	Male	3
50–59	5	Not employed—carer	2	Not stated	2
60–69	2	Not employed—other	5		
70	3	Not stated	2		
Not stated	2				

**Ethnicity**	***N***	**Disability**	***N***
White—British	9	Yes	5
Black African and White	1	No	6
Pakistani	3	Prefer not to say	2
Not stated	2	Not stated	2

The sample size was pragmatically decided (based on the team's capacity to support weekly calls). Diarists represented diversity in their circumstances (e.g., living alone or with families/partners and/or had caring responsibilities) and were purposefully sampled for their role as public advisers with research experience. One third of the participants were either shielding personally or living in a household with a family member who was shielding.

The first week of diary keeping commenced 4 weeks into England's first national lockdown and spanned the relaxation of rules in May 2020. Each diarist was teamed with a researcher (K. K., F. W., E. C. H., P. W.), who completed an induction and conducted subsequent weekly calls. All members of the research team were female and highly experienced in conducting social research. The diary template was designed in Qualtrics (online survey software), and an email link was sent weekly to diarists, who completed diaries for 8 weeks from 20 April to 14 June 2020. A Word version was available where participants preferred. The semistructured diary template combined closed questions asking participants to self‐rate their general and emotional health and quality of life with free‐text space to record daily activities as well as general reflections on their week. The self‐rated questions were mainly intended as ‘warm up’ questions before free‐text entries,^9^ but were also a useful analysis tool to understand how diarists reported emotional health week by week, and the factors influencing this. Similar to previous studies,^10^ we did not ask questions explicitly about the pandemic, allowing diarists to self‐direct what they chose to record. One exception occurred midway through the study, where diarists were prompted to reflect on a major change to national lockdown rules.

Weekly calls took place by video or telephone, lasting, on average, 30 min. A proforma guided the conversation, including prompts about diary keeping experiences and observations on diary entries. Calls were treated as research data with fieldnotes written up, but not audio‐recorded. Finally, an online focus group explored diarists' perspectives of the diary method. It was moderated by two female researchers (V. H., G. S.) not involved in the weekly calls, to enable participants to reflect more openly.

Research data including 115 diaries and 114 fieldnotes from weekly calls were imported to NVIVO‐12 (qualitative analysis software). Analysis was performed by two researchers (E. C. H. and V. H.). Thematic analysis in NVIVO‐12 initially identified prominent categories in the data set, drawing out similarities and differences.^27^ A key limitation of thematic approaches in analysing diary data is the potential to lose the nuances of ‘within person’ narratives over time.^16^ Therefore, the next stage of the analysis was informed by a narrative approach.^28^ Narrative tools outside of NVIV0‐12 included charting and textual summaries to explore mental health trajectories within and across weeks and key events and contextual factors featuring in these narratives.^29^ Analysis meetings with the research team built consensus about emerging themes and findings. Respondent validation took place at a workshop with diarists. Reporting was guided by the COREQ checklist^30^ and a framework intended to improve rigour in diary studies.^31^ Ethics approval was granted by Lancaster University Faculty of Health and Medicine's Ethics Committee in April 2020 (FHMREC19076). Written consent was obtained from diarists, who were also reminded of their rights during each weekly call. Processes were in place to support both the researchers and the diarists in the event of emotional distress, with weekly calls used to check in with diarists. All quotes and excerpts in the findings are anonymized using an identifier combining the diarist's unique ID (1–16) and the diary week (1–8). Where data come from weekly calls, this is stated (e.g., *diary5‐week1‐call*).

## 3 FINDINGS

### 3.1 Influences on mental health and well‐being

Four key analytic themes emerged as influencing mental health. These concerned risk of the virus, separation and loss of social connections, constrictions of the domestic space and the effects of the pandemic on diarists' sense of control. These were predominantly factors that posed risks to well‐being, but varied over time and in relation to diarists' situations.

#### 3.1.1 Navigating the risk of the virus

During the early weeks of the pandemic, receiving formal notification of being clinically vulnerable was anxiety provoking, particularly if an individual had not anticipated being in this group. This had caused one diarist to now feel ‘nervous about going out’ (diarist3‐week2‐call). For others with an existing appreciation of their risk levels, the latter still caused ‘significant anxiety’ [as] ‘it's been made clear it would be unlikely I would survive having it’ (diarist4‐week7). For those not shielding, navigating public spaces could also act as a major stressor, as the following quote illustrates:

Today I ventured out to do my big shop wish I had stayed at home had to queue for 1 hour and 20 minutes just to get into Asda. This triggered not one but 3 panic attacks the 3rd one when I was in the shop so didn't get what I need just needed to get out of shop and get home as quickly as possible (diarist6‐week1)

In some instances, it was only when a situation ended that there was realization about its psychological effects. Being involved in a community response was a positive experience for another diarist. Yet, this volunteering role was also highly stressful due to the responsibilities and risks posed.

Relieved, and happy that I (along with … friends and colleagues) have so far survived contracting the virus, despite placing ourselves (and our loved ones) in danger. The stress of being in close proximity with vulnerable residents, many of them elderly, with multiple and complex health conditions has been enormous at times. (diarist11‐week7)

Finally, changes in lockdown policy also left many participants concerned about rules being relaxed too soon. Diaries recorded apprehension among those who were previously happy to visit shops or public spaces during lockdown. For a participant living near the beach, for example, the arrival of ‘packed trains like sardines’ combined with busier shopping environments made them feel ‘more than a little anxious’ (diarist1‐week7‐call).

#### 3.1.2 Separation and loss of social connections

Fear for one's own situation was not an overriding narrative, but was frequently mentioned in relation to friends and family, a concern that was compounded by physical separation. At the start of diary keeping, diarist2 expressed considerable upset at being unable to support elderly parents living abroad, a situation that led to escalating anxiety as the situation worsened in their parents' country:

Emotionally I am not too good this week as the Coronavirus is bad … where my family is. So I am worried about my family. (diarist2‐week8)

Where families were dispersed geographically, many participants turned to technology and spoke of connecting with others as a ‘lifeline’. During lockdown, major life events were marked in this way such as birthdays or key life stages—‘had a couple of facetime messages—first one was my latest great granddaughter she has actually started to crawl great to be able to watch’ (diarist16‐week3) through to social activities and online services—‘inspired by the online worship and virtual coffee with church members providing positive connections and support’ (diarist5‐week6). Yet, the emotional distress of continued physical separations emerged repeatedly over the weeks. Diarists wrote of the pain of separation. For one person involved in their grandchildren's daily care, the ‘feeling of loss’ was described as a ‘physical ache in my chest’ (diarist8‐week1).

#### 3.1.3 Constrictions of the domestic space

While the domestic space was perceived largely as a safe space with respect to avoiding risk from the virus, confinement to home for large periods of time had varying implications for mental health depending on personal circumstances. For those shielding, mixed feelings were expressed about the thought of leaving their home after isolating. Some diarists were extremely apprehensive and said they would continue to isolate, whilst others were looking forward to returning to normal life. In a minority of cases, the time spent at home appeared to affect confidence about going out at all. Despite being regularly out of the house before the pandemic, diarist16 described in a weekly call that they no longer felt ‘safe’ leaving the house, attributing this to the length of time spent at home (diarist16‐week6‐call). In contrast, however, where diarists lived alone, spending long periods at home could be negative for emotional health as this diarist describes: ‘I realised early on during the lockdown that … 20+ hours in my own company in my own flat, not good’ (diarist10‐week1).

Carers faced a different set of stressors. They needed to navigate the dual tension of a more challenging home environment (if formal carer support had reduced), but there was also less opportunity to spend time away from the domestic space, previously a means of respite before the pandemic. For individuals with primary caring responsibilities, the situation created additional strain as a result of escalating roles, in the absence of additional carer support.

I'm carer, cleaner, gardener, chiropodist, barber, painter and decorator, IT consultant setting [parent] up on … Zoom, FaceTime, councillor and supporting [parent's] physical and deteriorating mental health and have received no offer of support or appreciation…(diarist9‐week5)

Alongside this, carers described stressors for family relationships, due to the amount of time spent together in a more stressful environment. When prompted about stress, one carer reflected that this stemmed from ‘probably just being locked up … and having to explain over and over again the situation [to the family member receiving support]’ (diary3‐week4‐call). Another diarist highlighted that the increased confinement to the domestic space also meant that other family members now had greater exposure to the reality of caring responsibilities, which could at times be distressing. They described the combination of several factors coalescing over the weeks, suggesting ‘it[‘s] just these small things that can gather up’ (diarist14‐week3‐call).

#### 3.1.4 Reducing autonomy and control

Where relatives and friends undertook shopping, or formal support was offered, this was highly appreciated by diarists. Yet, this support had consequences for the autonomy and control of those shielding. Particularly problematic was the conflation of health and social ‘vulnerabilities’ in the discourses surrounding risk groups. One diarist described being contacted by several agencies with offers of support. While this had initially felt reassuring, it increasingly affected how they related to their health identity, as ‘they [the agencies] all [are] reinforcing that fact that I'm a Shielded person and classing me as extremely vulnerable’ (diarist4‐week4). This left the individual feeling ‘more worried about my condition than I have felt for a very long time’ but also served as a ‘knock’ to their confidence. This issue was also evident when navigating previously routine activities such as grocery shopping. Another individual received support from a family member with shopping, but needed to consider alternatives when their daughter was unwell and it was difficult to obtain a supermarket delivery slot. However, as noted in a weekly call, the prospect of receiving local authority support also did not ‘feel right’ because ‘they only give food parcels’ (diarist16‐week3‐call).

### 3.2 Coping mechanisms

As well as showing the risks of the pandemic for mental health, diarists' accounts highlighted a range of strategies used to cope with the challenging and unfolding situation. These strategies were often linked to positive well‐being and experiences and included seeking support from social networks, engagement with social and natural environments, establishing normality, finding meaning and taking affirmative action.

#### 3.2.1 Help seeking and support

Personal networks (e.g., friends and family) as well as other social networks (e.g., volunteering, support groups, faith based) provided an important source of support for many diarists where these networks were in place. Connecting with friends and family or talking through difficulties had a positive impact on well‐being as the following quote illustrates: ‘Had a good chat with [relatives] and felt a lot better mentally’ (diarist1‐week12). Where diarists were connected into support group or faith networks, this provided a means of mutual support. In a weekly call, for instance, diarist14 explained that it had been good to meet up virtually with other carers in the same situation due to the ‘empathy they give you and the understanding of your situation’ (diarist14‐week4‐call).

Support was also instigated by friends and family as well as sought. Diarist6 described their children bringing a cup of tea and providing space when their mental health was poor. Similarly, diarist1 recalled how a friend had been ‘keeping an eye’ after receiving unwelcome news about their home situation. In this respect, the accounts illuminate how friends and families picked up on ‘signs’ when people were having a difficult day or week and took action accordingly.

A friend sent me a text … She then rang me after my short response which was unlike me. We had a good talk. (diarist5‐week3)

Unplanned events were also described, such as meeting a neighbour, which provided an impromptu opportunity to discuss how people external to the diarists' households were coping. Events that would not have been considered momentous before the pandemic also became symbolically more important. For instance, the delivery of shopping by family members became events to look forward to, and as restrictions lifted, offered the opportunity to physically see loved ones.

Formal help seeking was a minor theme in the findings. One diarist considered contacting their general practitioner following several weeks of deteriorating mental health. Two other diarists referred to mindfulness and self‐help techniques to maintain positive mental health. As noted earlier, however, in some situations, the lack of external support left people feeling isolated. Furthermore, where friends and family were facing additional pressures (e.g., juggling schooling and work) or volunteering opportunities had reduced, this could leave some diarists feeling disconnected from their routine networks.

#### 3.2.2 Engaging with social and natural environments

Opportunities to connect with social networks therefore acted as a key source of support for many diarists. Beyond this, a narrative also emerged about the importance of interactions with others within local communities as well as the environments around them. One diarist living alone described how they began to notice familiar faces on their walking route and feeling 'uplifted’ by people saying hello (diarist10‐week2‐call). This encouraged the diarist to start walking at regular times. Another diarist similarly wrote about the symbolic importance of ‘seeing the world’ after continued isolation. In 1 week, they intentionally chose a new walking route, which took them to a local road: ‘It was only cyclists who came by, but they waved and said a cheery hello’ (diarist4‐week3).

Positive benefits from interaction with the environment, particularly during the good weather, also featured in many diaries. Diarists outlined how going for walks or spending time in gardens, parks or other green spaces improved well‐being and helped them to unwind from daily pressures.

…straight into my garden, one coffee, one cigarette, and a beautiful hour, hosing, watering the sun beaten shrubs, bushes, trees, plants and veg… (diarist11‐week5)

Another diarist explained that being outside on a daily basis was an essential element of their routine. By taking a camera on daily walks, this helped ‘focus the mind’, which in turn contributed to an increased awareness and appreciation of their surroundings.

Everything in nature is so vibrant, and changes daily, there is always something new to hear/smell, observe every day and as I am in the park every day, I do notice it! (diarist10‐week3)

#### 3.2.3 Establishing normality and routine

Maintaining a routine both during the day and throughout the weeks emerged as a key narrative across diarists. During episodes of feeling low or depressed, diarists recorded disruption to their routine, leading to poorer sleeping patterns or a retreat from others or daily activities. During a weekly call, diarist1 explained how the week had begun well, but then ‘became depressed and had a couple of days where they stayed in, found it difficult to get out of bed or do anything much at all’ (diarist1‐week2‐call). In these situations, diarists recognized the importance of reintroducing routine, although this required self‐discipline: ‘myself and routines do not sit well, but it's important that I stick to it…so far its working’ (diarist10‐week5). Diarist1 similarly described the importance of daily walks, completing work‐related tasks and ensuring social contact with others in enabling them to regain positivity and purpose.

Worked hard this week to stay productive and positive feel I have achieved a lot this past week. (diarist1‐week3)

In this respect, setting and completing tasks, ranging from jobs in the home and garden, completing an artwork or finishing religion studies after several years, provided a sense of accomplishment. However, as diarist2 also noted, this did not necessarily need to be a significant event, rather ‘a lot of small things make a difference’. Similarly, the routine of activities (e.g., planning meals for the week or sorting out the home and garden) was frequently referenced, as such activities occupied minds and contributed to a sense of normality.

As lockdown restrictions were relaxed, there was some optimism about the return of normality and diarists focused on events to look forward to such as a reorganized family gathering or holiday. Largely, though, there was an overriding sense of caution and uncertainty. This stemmed from concern that lockdown rules were being relaxed too quickly, potentially resulting in a resurgence of the virus. For those who had been shielding, it could also feel hard to feel optimistic about continued shielding when others in the community were gaining their ‘freedom’.

#### 3.2.4 Taking affirmative action

Affirmative (positive) action was evident in relation to diarists' personal situations and the diarists' contributions to the pandemic response. First, at a personal level, among a minority of diarists, the situation focused attention on life planning such as ensuring that financial arrangements and wills were up to date. For many diarists shielding, the pandemic heightened attention to this issue, but for one diarist, the situation led to a ‘frank conversation’ about end‐of‐life wishes with a relative, which had not happened before the pandemic (diarist9‐week3). Nevertheless, while life planning was a way of taking control and diarists reported feeling reassured that their affairs were in order, such discussions could also generate ‘significant anxiety’ during a stressful time.

Second, many participating diarists were volunteers before the pandemic, and derived purpose and meaning from involvement in community action. Where possible, diarists continued routine volunteering where this transitioned online. Alongside this, the diaries and weekly calls highlighted several instances where diarists were directly involved in the pandemic response both through formal volunteering (e.g., for a befriending telephone service) and informally (e.g., setting up a fundraiser). Taking positive action was often described to benefit well‐being. The following diary entry reflects on a fundraising activity undertaken by a shielding diarist.

Have received messages [since the fundraiser] which have had a huge impact on how I have been made to feel. The more you put into life, the more you get out of it. So true. My week has reinforced this (diarist4‐week2)

Other ad hoc examples of positive actions were also observed. Diarist5 reported creating a video of a favourite hymn. This was shared with their church congregation for others to gain strength from (diarist5‐week7). For another diarist who enjoyed physical activity, coming up with an idea for an online classes made them ‘feel good’ for having suggested the idea to the fitness instructor (diarist14‐week3).

### 3.3 Finding meaning and sense making

In a minority of diaries, lastly, it was shown how sense making of the situation occurred through drawing on faith as well as past memories. In these diaries, entries included extracts from religious or uplifting texts, which were explained as providing comfort and strength to individuals. For some, the situation led diarists to reflect on those who had lived through other global events as a way of processing current events.

During this time I have thought a lot about her [mother] and my father and their generation, wondering what they would have made of it all and how they would relate it back to war times. I would love to have been able to talk it all through and understand more of what they faced and in particular how they faced the ongoing aftermath (diarist4‐week8)

In many cases, these recollections provided comfort. For example, diarist12 referred to the recollection of ‘fond memories’ triggered by a walk in their local area.

Not only went to [Greenspace 3] but also [Greenspace 4]…. both family and personal [memories] came flooding back and put me in a better place mentally (diarist12‐week8)

Yet, such memories were not always positive; for example, they could accentuate the grief and loss of family members who had died prematurely. For one diarist, the social effects of the pandemic also provoked parallels with past experiences that had resulted in a negative effect on their mental health at the time.

Finally, while the diaries were not intended as a therapeutic tool, we reflect next in the discussion, how the act of diary keeping impacted positively on some diarists' wellbeing. Alongside this, we reflect on the ethical issues associated with diary keeping during a pandemic.

## 4 DISCUSSION

This study contributes to an accumulating body of evidence highlighting the multiple mental health stressors associated with the pandemic such as social and physical isolation, challenging living environments, disruptions to normal routines and fear and insecurity.^32^, ^33^, ^34^ Strategies used by diarists to manage the immediate effects of the pandemic are also reflective of coping methods observed in other crisis contexts. Existing research, for example, has observed the role of social contact with others and supportive networks as well as the importance of maintaining regular habits and routines.^32^, ^35^, ^36^ Similarly, diarists' strategies demonstrated the ways in which individuals drew on strengths to cope with the unfolding situation. This reflects findings from Rodrigues and colleagues' recent review of social isolation among older adults during the pandemic, which showed individuals finding positivity in daily life, drawing on faith, learning new skills and adapting to online technology to maintain social relationships.^36^

While the mental health consequences observed during COVID‐19 are thought to share similarities to psychological effects observed in natural disasters (e.g., earthquakes, flooding), as well as previous pandemics such as Ebola,^33^ the sheer scale of mental health needs across local populations has particularly been noted.^34^ In this context, researchers have highlighted the need for mental health strategies that include community‐level action to foster social support and connectedness in local populations.^34^, ^37^ Yet, recent reviews show that the majority of available evidence on mental health interventions during crises is largely concerned with the needs of higher risk groups such as health care professionals, those quarantined by an infectious disease, children and young people and people with existing mental health conditions,^32^, ^37^, ^38^, ^39^ as well as individual‐level interventions such as psychological therapy, counselling or hotlines.^33^, ^37^, ^38^, ^39^, ^40^

Although this is a small‐scale study, our findings resonate with a need for a greater focus on community‐level strategies to protect mental health during crisis contexts. Many of the coping strategies observed within the diarists' responses shared a collective or social focus such as volunteering and community action, or were related to people's sense of belonging or identity to a community or place. Such dimensions of control, identity and belonging are protective for mental health in their own right,^41^ but could, arguably, also contribute to psychological preparedness in the context of future crises.^42^ Paradoxically, however, our study also showed that experiences of shielding and lockdown as well as official discourses surrounding vulnerability posed a risk to people's sense of autonomy, independence and confidence, with implications for well‐being. This finding reflects wider debates in relation to the extent to which public, media and professional discourses may undervalue the strengths and contributions of older adults or at‐risk groups during crisis periods.^43^ Finally, diarists' situations also showed vastly different experiences of the pandemic, highlighting that crisis responses also need to address underlying social determinants of mental health,^34^ rather than focusing only on building resilience of individuals and communities.^44^

A key limitation relates to the small participant sample, which largely reflected a female and older demographic who were retired or not in paid employment. This meant that the study did not address the implications of juggling work and family responsibilities, which has gender‐related implications for mental health.^45^ The sample also represented a group extensively connected into community and volunteering networks. While not a limitation necessarily, these experiences may not be reflective of the general population. Within the team, there was also variation in the extent to which diarists were known to the researchers, which may have had implications for the time that it took to build trust and what participants chose to disclose to individual researchers.

In general terms, diarists reported participation to be positive. Similar to other studies, diary keeping was described as therapeutic and offering emotional benefits,^14^, ^15^ as well as offering purpose, particularly for those shielding. Differences were recorded in how diaries were completed, with some participants writing more reflexively, whereas other diarists chose to record more factual information. Similarly, diary keeping and the weekly calls were experienced in different ways. In some cases, diaries recorded private feelings and emotions; however, other diarists preferred to reflect during weekly calls, describing them as an ‘emotional release’.

Challenges were observed with respect to the study's emotional and ethical aspects. More generally, some diarists expressed anxieties about confidentiality, also affecting what they chose to disclose. This required careful consideration of how sensitive information was presented, for instance, limiting the use of detailed individual narratives from diarists' data in the findings and presenting these more thematically.^16^ The study also raised specific implications for using diaries during a crisis. First, the unprecedented context that people were living through had implications for diary keeping due to time, family and emotional pressures within personal situations. Second, it became evident that for some diarists, the weekly calls offered an important form of social connection during lockdown. Although some onward opportunities for participation were in place, both diarists and researchers expressed a degree of concern that the end of the project occurred too suddenly. This raises implications more generally for the ethical responsibilities of research teams in concluding studies, where participants might be experiencing isolation.^15^ Lastly, the study was conducted in a relatively unusual context in that researchers were studying a situation that they were themselves affected by.^46^ In this respect, it could be challenging for researchers to listen to emotional experiences and upset. While reflexivity was considered as an ongoing process, of particular benefit was a training session convened with an independent counsellor before the study. This provided advice on maintaining researchers' emotional health as well as strategies for engaging during weekly contacts. This included, for example, making a conscious effort to not offer direct advice about a situation or share personal experiences in ways that may have influenced the nature of the data gathered.

## 5 CONCLUSION

This study is one of only a handful of qualitative diary studies conducted during a major societal crisis,^9^, ^10^, ^22^, ^23^ showing how the use of diaries can be beneficial in documenting lived accounts and emotional experiences of an ‘everchanging present’.^47^ The findings reveal the ways in which coping strategies adopted to cope with the pandemic's effects often had collective or social dimensions, which, in turn, could have benefits for well‐being. Nevertheless, the feasibility of this method during crisis periods will be dependent on the personal circumstances of those participating (e.g., the extent individuals have time to commit) as well as the nature of the crisis (e.g., the extent to which the crisis presents an immediate threat to people's lives and living circumstances). In particular, careful consideration is required of ethical implications in conducting crisis‐related research,^48^ particularly where the process of the research, such as diary keeping, may in itself have emotional consequences.

## CONFLICT OF INTERESTS

The authors declare that there are no conflict of interests.

## Data Availability

The data that support the findings of this study are available on request from the corresponding author. The data are not publicly available due to privacy or ethical restrictions.
